# Comparative analysis of periodontal pain and quality of life in patients with fixed multibracket appliances and aligners (Invisalign®): longitudinal clinical study

**DOI:** 10.1186/s12903-023-03565-z

**Published:** 2023-11-11

**Authors:** Alfonso Alvarado-Lorenzo, Laura Antonio-Zancajo, Hugo Baptista, Pedro Colino Gallardo, Alberto Albaladejo-Martinez, Daniele Garcovich, Silvia Alcon

**Affiliations:** 1 Department of Oral Surgery, Universidad de Salamanca, Salamanca, 37007 Spain; 2 Department of Dentistry, Universidad Católica de Murcia, Murcia, 30107 Spain; 3 Department of Dentistry, Universidad Europea de Valencia, Valencia, 46010 Spain

**Keywords:** Orthodontics, Pain, OHIP-14: location, Degree of pain, Invisalign.

## Abstract

**Backgrounds:**

The aim of this longitudinal clinical study is to analyse and compare according to location, degree and type, the pain presented by patients during their first year of treatment, as well as the quality of oral life after the placement of two types of orthodontic appliances: conventional brackets and removable Invisalign ® aligners.

**Methods:**

The sample consisted of 140 patients grouped into 2 study groups of 70 patients each. The first group (brackets group- BG), with fixed multibracket appliances, using the MBT technique and a 0.022” slot. The second group (invisaling group- IG), in treatment with removable aligners (IG), using the Invisalign ® system. They were providen with a questionnare where they had to record the degree (mild, moderate or intense), the type and location of the pain monthly during the first year of treatment. The second form was the Spanish version of the OHIP-14, oral quality of life questionnaire, which was provided the twelfth month after the start of treatment.

**Results:**

In both groups, we found that the most frequent location of pain occurred during the first phase: mandibular for the IG group and maxillary in the BG group. Throughout the whole analysis, the intensity was mild-moderate with lower values in the conventional brackets’ group. The BG group reported acute pain while the IG group reported sensitive pain during the first month; later both reported sensitive pain.

**Conclusions:**

There are differences in terms of periodontal pain in its degree, location, and type according to the different orthodontic techniques used.

**Trial registration:**

The study was approved by the bioethics committee of the University of Salamanca (USAL_20/516).

## Introduction

Much of orthodontic treatments are justified on the basis of improving psychosocial well-being and quality of life. The orthodontist has to provide information to patients about the needs and the results of the treatment, moreover he has to demonstrate responsibility regarding the effectiveness of the orthodontic treatment and the efficient use of resources [1].

Nowadays, patient’s perception is gaining more weight when evaluating the need for a treatment, its planning, and the established clinical aim. In addition, patients are aware of the importance of oral health and the impact that this may have on their lives, so they participate in the choice of the type of appliance to be used in their treatment. The repercussions on their quality of life will depend on their final choice, which will be largely determined by the degree of pain and discomfort presented during the treatment [2–6].

In general, the perception of pain will depend on the patients’ characteristics such as emotional factors, gender or age, as well as their ability to adapt to the situation. Here, aspects such as the way the patients face it or their character in stressful situations will influence. According to different studies, between a 90% and a 95% of the patients have reported discomfort or pain during their dental treatment [3, 7–11], although most of the time the pain was classified as mild and of short duration [4, 12–17]. Having pain at a previous visit to the dentist could cause a decrease in pain tolerance in the following appointments [18–20].

As has been observed in the different studies consulted, after the placement of orthodontic appliances, regardless of the technique used, the peak pain appears 24 h after the start of treatment and, subsequently, decreases [13, 14, 19, 21–25]. The pain usually has a dentogingival location, in most cases localized and rarely spontaneous, appearing after a functional stimulus such as chewing [7, 24, 26–28]. We can find, in the scientific literature, articles with results of lower levels of pain during the first week of treatment in patients treated with aligners [29–32]. White y cols. observed that pain in patients with conventional brackets or aligners was similar in the first two days of treatment, increasing, comparatively, in patients with conventional orthodontic later on [31]. In contrast, Cardoso y cols. found that patients with aligners experienced less pain in the early stages and they did not observed differences in the days after [33].

According to published studies, there are no gender differences in pain perception [6, 13, 17, 21]. However, differences have been observed in terms of age: adult patients notice pain more frequently, although the highest intensity is observed in teenager groups [8, 34–36].

It has been asserted that the level of osseodental discrepancy may affect the response to pain during the alignment process in orthodontic treatment, since the reduction of the interbracket space increases the stiffness of the arch and, as a result, it increases the forces expressed on the teeth [37]. Nevertheless, several studies have not shown a significant relationship between pain associated with applied orthodontic forces and the level of dental crowding [38–41].

The aim of this longitudinal clinical study was to analyse and compare the differences in terms of pain (location, type and degree of intensity) and the impact on the oral quality of life of patients treated both with multibracket fixed appliances and removable aligners, during the first 12 months of treatment. The null hypothesis of this study is that there are no statistically significant differences in the location, type and intensity of pain described by the patients as well as in the impact on their oral quality of life after treatment with conventional brackets and aligners.

## Material and method

### Study design

A clinical study was carried out in order to analyse pain perception in 140 patients both with fixed and removable orthodontics. All participants were informed about protocols, and they gave their written consent to participate in the study. The study followed the established guidelines by the Declaration of Helsinki for medical research involving human subjects and was approved by the bioethics committee of the University of Salamanca (USAL_20/516).

To estimate the sample size, previous research was carried out to calculate the sample size with an error rate of 5% and a confidence level of 95%. The minimum required sample size was considered to be 67 patients for each treatment group. Therefore, the final sample consisted of 140 participants. No patient dropped out of the study during the study. Study participants were selected using non-probability sampling, with efforts made to balance the sample in age and sex between each orthodontic treatment group. They were divided into two groups, all of them treated by the same operator throughout the study:

- A first group (BG) of 70 patients undergoing orthodontic treatment with multibracket fixed appliances, using MBT technique and a slot of 0.222” (Diamond Plus®, Cimbis Orthodontics, Madrid, España).
- A second group (IG) of 70 patients undergoing orthodontic treatment with removable aligners, using the Invisalign® system.

Study participants were selected using non-probability sampling, with efforts made to balance the sample in age and sex between each orthodontic treatment group. For the selection of participants, the following inclusion criteria were followed: adult patients over 18 years who had not received prior orthodontic treatment and with permanent dentition; patients with a negative tooth-size discrepancy (TSALD) between − 6 and − 2 mm in both arches; patients with no missing teeth except third molars; patients with skeletal class I or mild skeletal classes II and III (ANB between 0 and 5 degrees) and patients for whom interproximal reduction (IPR) between 0.1 and 0.5 mm per tooth were planned (in the treatment plan). They had to have a good oral health (dental and periodontal) and, a good general health (no previous systemic or mental illnesses such as anxiety or stress). The following exclusion criteria were followed: need for orthodontic surgical treatment; who were taking medication that influences on pain perception (analgesics, antidepressants and / or anticonvulsants); pregnant; and those patients treated with auxiliary orthodontic appliances.

### Clinical protocol and methodology

In the multibracket fixed appliance group, both arches were cemented the first day of treatment using a 0.014´´ thermal nickel titanium initial archwire CuNiTi (Bio Low Force®, Cimbis Orthodontics, Madrid, Spain) with 1.3 mm inner diameter elastic ligatures (3 M USA). Revisions were performed every month, changing the CuNiTi archwire section after 4 months (0.016” x 0.022”) and after 8 months (0.019” x 0.025”). In rotations of more than 25 degrees, 0.10” metal ligatures were placed.

In the case of patients treated with Invisalign®, after taking impressions with polyvinyl siloxane silicone (Aquasil, Denstply Sirona, USA), using plastic trays on a first visit and the revision/approval of the Clincheck. The aligners were manufactured and then placed during the second appointment according to the attachments prescribed in the treatment planning. Invisalign® patients changed their aligners every 15 days.

After the placement of the appliances, the patients received a questionnaire in which they should record monthly (during the first year of treatment): the location of the pain (posterior biarch, anterior biarch, posterior maxilla, anterior maxilla, posterior mandibular, and anterior mandibular); the intensity of pain (measured through the visual analogue scale with 0 being no pain and 10 being the maximum peak of pain.) and the type of pain (throbbing, shooting, stabbing, acute, cramping, piercing, burning, dull, heavy, sensitive, terrible, exhausting, nauseating, awful, and cruel). Patients were instructed about pain locations, pain intensity or types of pain.

The second questionnaire provided to the patients was the Spanish version of the OHIP-14 oral health-related quality of life questionnaire, which was completed the twelfth month after the start of treatment. The OHIP index (Oral Health Impact Profile) is an instrument to assess the impact of oral health on the quality of life of patients. The OHIP presents the ability to assess the frequency with which a person suffers difficulties in performing certain functions or daily activities due to oral disorders [42].

The pain questionnaire was given to the patients to fill out the first 7 days of each month. This was supervised at the monthly visit to the clinic and at the end of the treatment they filled out the OHIP-14 questionnaire. In both cases, the patients were monitored to verify that the questionnaires were being completed at the appropriate time as well as that they were understood and completed correctly by the patients.

### Statistical analysis

The software used for data analysis was the statistical package IBM SPSS 25. Techniques and statistical tests applied are described below. To describe the quantitative variables of the sample (sociodemographic and clinical characteristics), the usual descriptors were reported: mean, and standard deviation (measures of central tendency and variability), as well as line graphs for visual interpretation. In the case of categorical variables of nominal type, frequency and percentage tables were used, in addition to circle graphs. The differences in these variables between the two groups defined by the treatment factor (conventional brackets and Invisalign) were analysed to verify patient differences in these characteristics. To study the differences depending on the treatment group in the quantitative variables, the Student’s T test for independent samples was used. In the case of categorical variables, Pearson’s X2 test was used. In all cases, a 95% confidence level was considered.

## Results

### Characteristics of participants

The sample used consisted of 140 patients, 70 in each treatment group (braces and Invisalign aligners) with an average age of 29.36 years (± 9.80), ranging from 20 to 40 years old. In the BG group we find an average age of 26.97 years (± 7.23), being this average higher in IG (31.74 ± 11.39). Regarding gender, of the total sample, 68 were men (48.60%) and 72 women (51.40%). The average of total upper bone-dental discrepancy was − 3.34 (± 1.86) and in the lower arch, -3.42 ( ± = 1.54). We find, therefore, a homogeneous sample in terms of age, gender and bone-dental discrepancy.

### Analysis of the intensity and dentoperiodontal location of pain

After analysing the results, it is noted that the average pain level throughout the months of treatment is higher in IG, except for the first month, where BG reports a higher degree of pain. It should be noted here that the IG standard deviation is larger in all cases, indicating that there is greater variability in the degree of referred pain within participants in this group. The highest degree of pain is experienced in the first month of treatment in both groups (BG: 2.175 ± 1.98; IG 1.976 ± 2.295). From that moment, the pain starts to decrease progressively, month by month, finding an increase in pain in the BG group during the eighth month of treatment (0.57 ± 0.962). For IG this decrease is less pronounced, maintaining a higher level of pain from the second month, compared to BG, and some higher peaks taking place in the sixth (0.926 ± 1.510) and tenth (0.740 ± 1.510) months, compared to that decreasing trend that was found from the first month (Table 1).

**Table 1:** Comparison of pain between both treatment groups over 12 months (n = 140)

Month	Conventional Braces (*n =* 70)		Invisalign^®^ (*n =* 70)		
	Mean	SD	Mean	SD	*p*
**1**	2.175	1.984	1.976	2.295	0.097^*^
**2**	0.869	1.159	1.288	1.835	0.000^**^
**3**	0.642	1.009	1.197	1.904	0.000^**^
**4**	0.607	1.004	0.998	1.675	0.001^**^
**5**	0.405	0.792	0.884	1.483	0.000^**^
**6**	0.430	0.852	0.926	1.510	0.000^**^
**7**	0.331	0.731	0.791	1.442	0.000^**^
**8**	0.573	0.962	0.691	1.369	0.291
**9**	0.386	0.807	0.627	1.318	0.038^*^
**10**	0.380	0.761	0.740	1.510	0.002^**^
**11**	0.269	0.657	0.525	1.246	0.028^*^
**12**	0.202	0.580	0.452	1.145	0.031^*^

**SD**: Standard deviation. Comparisons between groups applying the **Student’s T test**. *: statistically significant differences with *p* < 0.05. **: statistically significant differences with *p* < 0.01

During the whole observation period, we noted both maxillary and mandibular anterior pains as the predominant pains of patients in both groups, with values between 70% and 85% depending on the time point. The posterior locations (maxillary or mandibular) and both (anterior and posterior maxillary or mandibular) had less incidence, not exceeding 26%. We noticed that in the maxillary anteroposterior location there are differences in terms of percentage of patients in both groups, finding that patients in the IG group presented a higher percentage of pain during the first months (coming to a difference of almost 11% points in the third month: 11.7% in BG and 22.4% in IG). These differences are decreasing in the following months. In the mandibular arch, this discrepancy was not observed in the anterior (most posterior) location in both groups (Table 2). The frequencies of location in the posterior area, in both groups, were always lower than those in the anterior area. In addition, regarding to the posterior areas, we find, again in both groups, percentages with higher values in the mandibular arch, being these values higher in IG in all months of the study.

**Table 2:** Frequencies of the location of pain according to the treatment group throughout the 12 months (n = 140)

Month	Group	Location of pain						
		Maxillary				Mandibular		
		Anterior N (%)	Posterior N (%)	Both N (%)	Anterior N (%)		Posterior N (%)	Both N (%)
1^*^	BG	303 (77.3)	20 (5.1)	69 (17.6)	299 (74.2)		27 (6.7)	77 (19.1)
	IG	252 (70.8)	13 (3.7)	91 (25.6)	216 (72.0)		38 (12.7)	46 (15.3)
Chi: 7.48, gl: 2, *p* value: 0.024 Chi: 8.14, gl: ,2 *p* value *p*: 0.017								
2	BG	195 (79.9)	14 (5.7)	35 (14.3)	195 (81.3)		16 (6.7)	29 (12.1)
	IG	240 (77.4)	12 (3.9)	58 (18.7)	166 (75.1)		25 (11.3)	30 (13.6)
Chi: 2.67, gl: 2, *p* value: 0.263 Chi: 3.55, gl: 2, *p* value: 0.170								
3^**^	BG	153 (85.0)	6 (3.3)	21 (11.7)	151 (81.2)		9 (4.8)	26 (14.0)
	IG	202 (72.9)	13 (4.7)	62 (22.4)	143 (71.9)		24 (12.1)	32 (16.1)
Chi: 9.43, gl: 2, *p* value: 0.009 Chi: 7.23, gl: 2, *p* value: 0.027								
4^*^	BG	133 (84.2)	5 (3.2)	20 (2.7)	144 (81.8)		13 (7.4)	19 (10.8)
	IG	164 (80.0)	17 (8.3)	24 (11.7)	109 (69.4)		26 (16.6)	22 (14.0)
Chi: 4.13. gl: 2, *p* value: 0.127 Chi: 8.34, gl: 2, *p* value: 0.015								
5	BG	105 (79.5)	8 (6.1)	19 (14.4)	89 (72.4)		17 (13.8)	17 (13.8)
	IG	169 (76.1)	17 (7.7)	36 (16.2)	129 (76.3)		19 (11.2)	21 (12.4)
Chi: 0.60. gl: 2, *p* value: 0.740 Chi: 0.64, gl: 2, *p* value: 0.726								
6	BG	97 (78.2)	4 (3.2)	23 (18.5)	93 (76.2)		10 (8.2)	19 (15.6)
	IG	153 (74.6)	13 (6.3)	39 (19.0)	135 (79.9)		18 (10.7)	16 (9.5)
Chi: 1.59, gl: 2, *p* value: 0.451 Chi: 2.76, gl: 2, *p* value: 0.251								
7^**^	BG	86 (81.9)	1 (1.0)	18 (17.1)	76 (75.2)		12 (11.9)	13 (12.9)
	IG	137 (74.9)	20 (10.9)	26 (14.2)	114 (76.0)		24 (16.0)	12 (8.0)
Chi: 9.91, gl: 2, *p* value: 0.007 Chi: 2.16, gl: 2, *p* value: 0.340								
8^**^	BG	108 (76.1)	7 (4.9)	27 (19.0)	132 (78.6)		11 (6.5)	25 (14.9)
	IG	128 (76.2)	13 (7.7)	27 (16.1)	97 (69.8)		28 (20.1)	14 (10.1)
Chi: 1.32, gl: 2, *p* value *p*: 0.516 Chi: 13.24, gl: 2, *p* value: 0.001								
9^*^	BG	92 (81.4)	0 (0.0)	21 (18.6)	92 (13.7)		6 (5.2)	17 (14.8)
	IG	115 (76.2)	11 (7.3)	25 (16.6)	91 (75.2)		16 (13.2)	14 (11.6)
Chi:8.61, gl: 2, *p* value: 0.013 Chi: 4.69, gl: 2, *p* value: 0.096								
10^**^	BG	107 (84.3)	2 (1.6)	18 (14.2)	78 (75.0)		7 (6.7)	19 (18.3)
	IG	101 (67.8)	19 (12.8)	29 (19.5)	97 (68.8)		22 (15.6)	22 (15.6)
Chi: 14.85, gl: 2, *p* value: 0.000 Chi: 4.56, gl: 2, *p* value: 0.102								
11^*^	BG	71 (82.6)	2 (2.3)	13 (15.1)	73 (82.0)		5 (5.6)	11 (12.4)
	IG	95 (79.8)	6 (5.0)	18 (15.1)	79 (72.5)		20 (18.3)	10 (9.2)
Chi: 0.99, gl: 2, *p* value: 0.610 Chi: 7.34, gl: 2, *p* value: 0.025								
12	BG	57 (82.6)	1 (1.4)	11 (15.9)	45 (76.3)		5 (8.5)	9 (15.3)
	IG	87 (81.3)	6 (5.6)	14 (13.1)	65 (69.9)		17 (18.3)	11 (11.8)
Chi: 2.07, gl: 2, *p* value: 0.309 Chi: 2.92, gl: 2, *p* value: 0.232								

**BG**: conventional braces. **IG**: Invisalign**®**. ^*^: statistically significant differences with *p* < 0.05. **: with p < 0.01 in, at least, one of the locations (maxillary or mandibular).

### Analysis of the pain scores by groups at different time points

Statistically significant differences are found with p < 0.01 in the groups in each of the months analyzed. In the first month of treatment, the group that reported less intensity of pain was BG with lower percentages than IG (82.9% vs. 71.2%). This situation is repeated every month of the study. We also observed that the frequencies in the expression of intense pain disappeared in BG from the second month of treatment (0%). In the case of IG, high figures remain, on the one hand, in moderate pain and lower figures in severe pain, but always higher than those of BG. This shows that pain is always greater in IG (Table 3).

**Table 3:** Frequencies of pain intensity according to the treatment group during the 12 months (n = 140)

Month	Type of treatment						
	Conventional Braces				Invisalign®		
	Mild N (%)	Moderate N (%)	Severe N (%)	Mild N (%)		Moderate N (%)	Severe N (%)
1^**^	389 (82.9)	74 (15.8)	6 (1.3)	326 (71.2)		123 (26.9)	9 (2)
Chi: 20.21, gl: 4, *p* value: 0.000							
2^**^	278 (98.2)	5 (1.8)	0 (0.0)	297 (77.5)		81 (21.1)	5 (1.3)
Chi: 60.20, gl: 3, *p* value: 0.000							
3^**^	223 (97.0)	7 (3.0)	0 (0.0)	266 (78.2)		68 (20.0)	6 (1.8)
Chi: 39.64, gl: 2, *p* value: 0.000							
4^**^	218 (99.1)	2 (0.9)	0 (0.0)	203 (74.6)		63 (23.2)	6 (2.2)
Chi: 60.86, gl: 4, *p* value: 0.000							
5^**^	158 (98.8)	2 (1.2)	0 (0.0)	236 (82.8)		43 (15.1)	6 (2.1)
Chi: 25.71, gl: 4, *p* value: 0.000							
6^**^	150 (98.0)	3 (2)	0 (0.0)	209 (78)		53 (19.8)	6 (2.2)
Chi: 31.26, gl: 2, *p* value: 0.000							
7^**^	136 (99.3)	1 (0.7)	0 (0.0)	203 (81.5)		40 (16.1)	6 (2.4)
Chi: 27.61, gl: 3, *p* value: 0.000							
8^**^	183 (96.3)	7 (3.7)	0 (0.0)	172 (81.5)		33 (15.6)	6 (2.8)
Chi: 20.20, gl: 2, *p* value: 0.000							
9^**^	139 (95.9)	6 (4.1)	0 (0.0)	173 (86.1)		23 (11.4)	5 (2.5)
Chi: 9.86, gl: 2, *p* value: 0.007							
10^**^	151 (99.3)	1 (0.7)	0 (0.0)	172 (84.3)		27 (13.2)	5 (2.5)
Chi: 23.41, gl: 2, *p* value: 0.000							
11^**^	111 (99.1)	1 (0.9)	0 (0.0)	138 (84.1)		24 (14.6)	2 (1.2)
Chi: 16.89, gl: 2, *p* value: 0.000							
12^**^	81 (98.8)	1 (1.2)	0 (0.0)	124 (83.8)		22 (14.9)	2 (1.4)
Chi: 12.26, gl: 2, *p* value: 0.002							

^**^: statistically significant differences with *p* < 0.01

### Analysis of the type of pain by groups and time point

After analyzing the types of pain reported by the participants, we find that the most described type of pain, in both groups, is sensitive pain (43.5% in BG and 48.6% in IG), followed by throbbing pain ( 28.7% in BG and 29.0% in IG) and acute pain (23.9% in BG and 16.6% in IG). The rest of the types of pain (shooting, cramping, piercing, burning, dull, terrible, exhausting, nauseating, awful, and cruel) were grouped together in the “other types” section (Table 4) due to their low incidence.

**Table 4:** Global percentage frequencies of the type of pain according to the treatment group (n = 140)

	Conventional Braces (*n = 70*)	Invisalign® (*n = 70*)	Total (*n = 140*)
**Throbbing**	28.7%	29.0%	28.9%
**Stabbing**	1.3%	2.4%	2.0%
**Acute**	23.9%	16.6%	19.6%
**Heavy**	1.9%	1.7%	1.8%
**Sensitive**	43.5%	48.6%	46.5%
**Other types**	0.7%	1.7%	1.3%

In the first month of treatment for both groups, the most frequent pain in the BG group was acute pain (46.7%) and, in the case of IG group, sensitive pain (37%). In the remaining months, in both groups, the most recurrent pain was sensitive pain, with percentages that vary between 40.6% (BG group during the second month) and 55% (IG group during the sixth month). We find it remarkable that after twelve months of treatment, patients still report a high percentage of sensitive pain in both groups, being 54.6% in the BG group and 52.0% in the IG group (Table 5).

**Table 5:** Frequencies of the type of pain according to the treatment group throughout the 12 months (*n* = 140)

**Month**		**Group**	**Types of pain**						
			**Throbbing** N (%)	**Stabbing** N (%)	**Acute** N (%)	**Heavy** N (%)	**Sensitive** N (%)	**Others** N (%)	
1*^*^		BG	71 (15.1)	5 (1.1)	219 (46.7)	1 (0.1)	164 (35.0)	9 (2)	
		IG	115 (25)	15 (3.3)	128 (27.8)	11 (2.4)	170 (37.0)	21 (4.5)	
Chi: 72.30. gl: 9, *p* value: 0.000									
2^**^		BG	83 (29.3)	7 (2.5)	78 (27.6)	0 (0.0)	115 (40.6)	0 (0.0)	
		IG	114 (29.8)	13 (3.4)	62 (16.2)	9 (2.3)	180 (47)	5 (1.4)	
Chi: 22.32. gl: 7, *p* value: 0.002									
3^*^		BG	67 (29.1)	1 (0.4)	54 (23.5)	4 (1.7)	104 (45.2)	0 (0.0)	
		IG	110 (32.2)	8 (2.3)	45 (13.2)	10 (2.9)	162 (47.4)	7 (2.1)	
Chi: 19.22, gl: 8, *p* value: 0.014									
4^**^		BG	39 (17.7)	3 (1.4)	58 (26.4)	6 (2.7)	111 (50.5)	3 (1.4)	
		IG	76 (27.9)	7 (2.6)	46 (16.9)	4 (1.5)	134 (49.3)	5 (1.9)	
Chi: 20.18, gl: 8, *p* value: 0.003									
5		BG	49 (30.6)	0 (0.0)	28 (17.5)	1 (0.6)	82 (51.2)	0 (0.0)	
		IG	81 (28.3)	5 (1.7)	42 (14.7)	2 (0.7)	150 (52.4)	6 (2)	
Chi: 6.90. gl: 7, *p* value: 0.440									
6^**^		BG	65 (42.5)	2 (1.3)	16 (10.5)	4 (2.6)	64 (41.8)	2 (1.3)	
		IG	67 (24.1)	5 (1.8)	48 (17.3)	4 (1.4)	153 (55.0)	1 (0.4)	
Chi: 22.45, gl: 6, *p* value: 0.001									
7		BG	45 (32.8)	2 (1.5)	31 (22.6)	12 (8.8)	46 (33.6)	1 (0.7)	
		IG	82 (31.5)	0 (0.0)	48 (18.5)	7 (2.7)	122 (46.9)	1 (0.4)	
Chi: 17.73, gl: 6, *p* value: 0.007									
8^**^		BG	73 (38.4)	2 (1.1)	32 (16.8)	0 (0.0)	10 (43.7)	0 (0.0)	
		IG	71 (32.3)	0 (0.0)	31 (14.1)	2 (0.9)	111 (50.5)	5 (2.4)	
Chi: 10.94, gl: 7, *p* value: 0.141									
9^**^		BG	56 (38.6)	7 (4.8)	14 (9.7)	4 (2.8)	64 (44.1)	0 (0.0)	
		IG	60 (29.9)	0 (0.0)	33 (16.4)	2 (1)	104 (51.7)	2 (1)	
Chi: 18.43. gl: 6. *p* value: 0.005									
10^*^		BG	59 (38.8)	0 (0.0)	15 (9.9)	5 (3.3)	73 (48)	0 (0.0)	
		IG	59 (28.8)	6 (2.9)	22 (10.7)	1 (0.5)	115 (56.1)	2 (1)	
Chi: 16.96. gl:7. *p* value: 0.018									
11		BG	37 (32.7)	2 (1.8)	7 (6.2)	2 (1.5)	65 (57.5)	0 (0.0)	
		IG	46 (27.9)	13 (7.9)	17 (10.3)	3 (1.8)	86 (52.1)	0 (0.0)	
Chi: 6.84. gl: 4. *p* value: 0.144									
12^*^		BG	26 (31.7)	0 (0.0)	6 (7.3)	5 (6.1)	45 (54.6)	0 (0.0)	
		IG	54 (36.5)	6 (4.1)	11 (7.4)	0	148 (52)	0 (0.0)	
Chi: 12.77. gl: 4, *p* value: 0.012									

**BG**: conventional braces. **IG**: Invisalign. ^*^: statistically significant differences with *p* < 0.05. ^**^: with *p* < 0.01.

### Assessment of the impact on quality of life of patients according to OHIP-14 questionnaire

We find statistically significant differences with ***p*** < 0.01 at the level of functional limitation, physical disability and obstacles. We noted that the maximum score of both groups in the OHIP-14 items concerns to pain (BG: 2.79 ± 1.605; IG: 2.43 ± 1.584). The second item with the highest score in BG is physical disability with a score of 1.31 ± 0.941, while in IG concerns to functional limitation with a score of 2.27 ± 1.693. Statistically significant differences in the dimensions of pain, psychic discomfort, psychological disability and social disability are not found. We can affirm that in all items (functional limitation (1.13 ± 1.115), psychological disability (0.37 ± 0.571), social disability (0.46 ± 0.582) and obstacles (0.03 ± 0.168)) the group that presented the least impact is BG. BG obtained higher scores in pain (2.79 ± 1.605), psychic discomfort (1.03 ± 1.103) and physical disability (1.31 ± 0.941). IG has a greater impact by its total values (7.43 ± 0.652) The IG has a greater impact due to its total values ​​(7.43 ± 0.652) compared to the bracket group but there is no statistically significant difference. However, in the total OHIP there are no statistically significant differences (Table 6).

**Table 6:** OHIP-14 items score by treatment group (n = 140)

	Conventional Braces (*n =* 70)		Invisalign® (*n =* 70)		
	Mean	SD	Mean	SD	*p*
**Funtional Limitation** ^**^	1.13	1.115	2.27	1.693	0.000
**Pain**	2.79	1.605	2.43	1.584	0.187
**Psychic Discomfort**	1.03	1.103	0.67	1.172	0.063
**Physical Disability** ^**^	1.31	0.941	0.81	1.081	0.004
**Psychological Disability**	0.37	0.571	0.46	1.099	0.548
**Social Disability**	0.46	0.582	0.70	0.922	0.065
**Obstacles**	0.03	0.168	0.26	0.630	0.004
**Total OHIP**	6.99	3.300	7.43	0.652	0.559

Comparisons between groups applying the Student’s T test. *: statistically significant differences with *p* < 0.05. **: with *p* < 0.01. SD: standard deviation

## Discussion

This longitudinal clinical study, during the first 12 months of treatment, aims to analyse and compare the degree, location and type of dento-periodontal pain between two types of appliances: fixed (conventional brackets) and removable (aligners). The follow-up time of the patients in this study, in terms of pain, is longer compared to other published studies [4, 21, 29, 30, 43–45]. The evaluation of the oral quality of life of the patients in our study was carried out by submitting the questionnaire in month 12 from the start of treatment. No published study has done this at that time, most do it within the first month [43, 46–49].

To produce the movement of the teeth in orthodontic treatments we apply, either through brackets and arches or through the aligners, we use various forces that if they are very high (more than 20 g) could cause pain in the teeth [14, 50, 51]. The degree of force received by the teeth can be increased in cases of large osseodental discrepancies: the greater the crowding, the greater the force received and the greater the pain suffered [15, 50, 52]. It has been asserted that the degree of discrepancy can affect the response to pain during the alignment process, since the reduction of the interbracket space increases the rigidity of the arch and, as a consequence, the forces expressed in the teeth [38]. Several studies have not shown a significant connexion between pain associated with applied orthodontic forces and the degree of crowding [38–41, 53]. To our knowledge, no published studies of these characteristics have been found that analyse the relationship between the degree of crowding and location (upper or lower), comparing fixed and removable (aligners) orthodontic techniques between them.

We have not found statistically significant differences, in relation to the gender of the patients, in the level of pain or the impact on their quality of life. In our study the gender ratio is very similar, with 35 men and 33 women in the conventional brackets group (BG) and 35 men and 37 women in the Invisalign® group (IG). Several studies with similar results have been found in which there are no statistically significant differences in the level of pain perceived by men compared to women [54–56]. However, there are other studies that state that women report more discomfort than men [50, 56, 57].

Regarding age, the selected patients were adults with an average of 26.97±-7.23 in the fixed appliance group and an average of 31.74 ± 11.39 in the aligners group. The patients were in permanent dentition and gender distribution was very similar: 68 were men (48.60%) and 72 women (51.40%). We find in our study a statistically significant difference with a *p* < 0.004 in aged [58]. However, this data is not considered clinically relevant, since all the patients were in permanent dentition. It is difficult to assess the aspect of age in pain perception. Most of the authors consulted consider that adult patients perceive pain more frequently than minor patients [8, 35, 37, 59].

After the scientific literature review, we find that most of the authors refer to dentogingival pain with fixed orthodontics and other types of appliances, however, they do not specify its location or focus their study on the comparison of dental pain versus mouth soft tissues pain (tongue, oral mucosa, lips) [33, 43, 52, 60]. In our study we aim to analyze dentoperiodontal pain at different time points during the first year of treatment. After the placement of the appliances we found that, in both groups, the predominant pain location was the anterior, both maxillary and mandibular. These results match with those obtained by other authors who consider that there is greater pain in anterior than in posterior teeth, in treatments with fixed appliances [8, 41, 61] and, especially, in the mandibular area [45, 62], unlike the results of our study where the anterior maxillary location pain is more predominant in the group of fixed appliances with a maximum value of 85% in the third month. Although the pain is less in the anterior area, aligners could create a dilemma in the anterior aesthetic area [63]. The posterior maxillary location obtained the lowest percentage (1%) in the fixed appliances group in the seventh month. In the case of IG, we found a greater pain in the anterior mandibular area with a maximum value of 82% in the eleventh month, what matches with the results obtained by other authors [64]. The posterior maxillary location also obtained the lowest percentage (5%) in this group in the eleventh month.

The pains most frequently described by the patients were acute, sensitive and throbbing, being the acute type the most frequent in the brackets group (46.7%), unlike other authors who find that they were throbbing [44, 45] or sensitive types [64]. In the Invisalign group, we find that the most frequent type of pain was sensitive (37%); other authors consulted reached the same results [44, 45, 64]. Our study matches with the study by González-Sáez y cols. [45] in terms of the types of pain most frequently described by patients (acute, sensitive and throbbing), and not only with this study but also with that of Curto y cols [44] in terms of the description of sensitive pain as the most recurring in IG. However, our study differs from the both mentioned since these authors [44, 45] affirm that the most frequent type of pain in BG is throbbing pain and in our study we find that it was acute.

Regarding the degree of pain that patients present during the first year of treatment, we find statistically significant differences in all analysed months. The pain was milder in the brackets group patients compared to those in the aligners group. As in the previous data, we can only compare our results with other studies during the first month of treatment due to the absence of data in the rest of months. Most of the authors consulted find that the degree of pain, with the various orthodontic appliances, moves, during the first week of treatment, in values of a mild-moderate nature [58, 64, 65]. We find these same results during the twelve months of analysis, where the pain was decreasing in degree as the treatment progressed, especially, in the fixed orthodontic group (Table 2). Pereira y cols [66], as other consulted authors, found in their 2020 meta-analysis that aligner patients present less pain than conventional fixed orthodontic patients [21, 33, 67, 68], however, it must be taken into account that their samples were smaller in most cases and they only analysed the first week of treatment. It is important to point that small sample sizes may cause confusion, since the smaller the sample is, the more imprecise the results will be found (given that the confidence intervals of the studied parameters will be broader). For this reason, we have previously calculated the sample treatment per group, which in our case is 67.

Discomfort during orthodontic treatment and its impact on oral quality of life are worse in the early phases of treatment and they gradually reduce as the treatment progresses until the final phase [48, 68–71]. Regarding the evaluation of quality of life through the OHIP-14 questionnaire, we find statistically significant differences with *p* < 0.01 in the items of functional limitation and obstacles (with a higher score for the group of aligners), and in physical disability item (with a higher incidence in the braces group). Azaripour y cols [72] assertes, in their study carried out with 100 patients, that patients treated with aligners had a greater satisfaction than those treated with fixed appliances, unlike our results where we find that, for the total OHIP, Invisalign patients reported higher total values (7.43 vs. 6.99), although the results were not statistically significant. Other authors also find greater total satisfaction in patients with aligners, compared to patients with fixed orthodontics [21, 73, 74].

In this study, we protocolized a follow-up time for patients of 12 months. This is the novelty of our study compared to other published studies: pain monitoring during the first year of treatment. The follow-up time of the patients in this study, in terms of pain, is higher than in other published studies, since most of them just analyse the period between the first and fifth week after the start of treatment [4, 21, 30, 43–46, 49, 52]. Regarding quality of life, we find in the literature that follow-up times go from the first 24 h of analysis to the total orthodontic treatment, although most of the measurements were made after one month of treatment [21, 43, 47, 48, 75–79].

One of the main limitations of this study was the sample in reference to the age of the patients, since the study was carried out only in adult patients (between 20 and 40 years old). In the scientific literature, there are studies that conclude that there are no statistically significant differences in the perception of pain when analysing the influence of age [8, 22, 35, 47, 80]. However, we consider that it would be convenient to compare it with different age ranges. It would also be convenient to increase the sample size in future studies and to evaluate different systems of brackets and aligners, as well as the influence of the consumption of analgesic and/or anti-inflammatory medicines during orthodontic treatment.

On the other hand, we also consider a limitation in the results of this study, the difficulty in randomization regarding the type of treatment for each patient. In this type of treatment it is difficult to randomize the sample since there are patients who cannot be forced which treatment to have (for example, having braces when they only want a removable treatment). Carrying out the study on a non-randomized sample, although both treatments are effective in resolving malocclusion, may have its limitations when it comes to assessing the results of pain intensity or quality of life, so in the future it would be ideal to perform it on a randomized sample.

## Conclusions

After monitoring the patients during the first twelve months of treatment, we find that the patients in the group of conventional brackets (BG) presented a lower degree of pain than the group of aligners (IG) in all analysed periods, although both moved between mild and moderate pain throughout the analysis time. This pain was located mainly in the anterior bimaxillary area (anterior mandibular in IG and anterior maxillary for BG).

The most frequently experienced types of pain were acute for BG and sensitive for IG during the first month of treatment. Subsequently, for both groups, the sensitive type was referred with a higher percentage.

Regarding quality of life, in the total OHIP, no statistically significant differences were found between the analyzed groups.

## Data Availability

The datasets used and/or analysed during the current study available from the corresponding author on reasonable request.

## Abbreviations

BG: Brackets group
IG: Invisaling group
OHIP-14: Oral Health Impact Profile − 14
TSALD: tooth-size discrepancy
IPR: interproximal reduction
CuNiTi: Cooper niquel titanium archwire
